# The Impacts of COVID-19 on Distance Education with the Application of Traditional and Digital Appliances: Evidence from 60 Developing Countries

**DOI:** 10.3390/ijerph19116384

**Published:** 2022-05-24

**Authors:** Jiajia Li, Shiyu Yang, Changju Chen, Houjian Li

**Affiliations:** College of Economics, Sichuan Agricultural University, Chengdu 611130, China; jiajia_li108@hotmail.com (J.L.); shiyu_yang2022@163.com (S.Y.); changju_chen48707@163.com (C.C.)

**Keywords:** COVID-19, distance learning, education inequality, lockdown measures, High Frequency Phone Survey, developing countries

## Abstract

Educational disruptions from the COVID-19 pandemic during school closures have become a remarkable social issue, particularly among the developing countries. Ample literature has verified the adverse effects of the long-lasing epidemic on school education. However, rare studies seek to understand the association between the severity of COVID-19 and distance learning, an alternative education pattern, and foster policy designs to promote educational transition, particularly targeting the post-crisis phase of the COVID-19. By combining four data surveys, this article empirically examines the impacts of COVID-19 on children’s distance education with the application of various appliances across 60 developing countries. The results suggest that, after controlling socio-economic, geographic, and demographic variables, a higher level of mortality rate of COVID-19 contributes to more households participating in distance education. In particular, this positive term is larger for distance education by using TVs and radios compared with the usage of digital appliances. To explore the potential channel of the above linkage, this article argues that the positive association between mortality rate and the use of traditional appliances is weakened through higher levels of stringency in lockdown measures. Timely policies are, therefore, recommended to guide towards distance learning with economic and technological supports to guarantee a wave of inclusive educational recovery in the ongoing post-COVID-19 era.

## 1. Introduction

COVID-19 is a respiratory disease that is caused by the novel coronavirus SARS-CoV-2, which is transmitted by droplets, close contact, and aerosols. SARS-CoV-2 was first identified in Wuhan, China, in December 2019, and was declared a global pandemic by the World Health Organization (WHO) on the 11 March 2020 [1]. The onset of COVID-19 has become one of the major events of global concern in 2020 and even in the coming years. As of 17 March 2022, the world has reported 462,758,117 confirmed cases and 6,056,725 deaths according to the WHO [2]. The estimated mortality rate during 32 days was 1.1% for the non-severe patients and up to 32.5% among the severe cases [3]. Remarkably, this global pandemic has been sustained for more than two years, which gives rise to further concerns of its longer-term hazards in the post-crisis phase towards different aspects of human’s wellbeing, particularly education.

Numerous studies have confirmed that the epidemic and its relative lockdown measures brought educational systems across the world to a halt [4,5,6]. At the peak of the pandemic, temporary school closures in more than 180 countries lead to 1.6 billion students out of school [7]. Hence, it is necessary to explore new educational patterns to alleviate the negative impacts of the long-lasting epidemic on education and to further guarantee the quality of millions of children’s human capital growth. During school closures, offline education was progressively replaced by distance education, at least in some developed countries [8]. Due to the long-lasting pandemic, many countries have to turn to distance education by employing a wide variety of applications, i.e., internet, computers, TVs, and radios, to deal with the disadvantaged education of the schools shutting down [9,10]. However, due to social-economic ignorance and governmental lockdown measures, the implementation of distance education in each country is quite different. Taking the emerging markets and developing countries as an example, there is a greater dilemma in the transformation from formal to distance educational modes during the epidemic due to the lack of educational funds, advanced technologies, and social supports [6,11,12,13,14]. Thus, greater attention needs to be paid to educational transformation, particularly in developing countries under the COVID-19 crisis.

As well known, the negative effects of the pandemic on formal education have been well explored in the literature, i.e., see Di Pietro et al. [15]; Schleicher [16]; Mustafa [5]. However, the associations between the severity of COVID-19 and distance learning are comparatively rare and this topic has greater implications to deal with the long-lasting worldwide coronavirus shock from the perspective of supporting worldwide education equality. Moreover, even fewer studies explored the underlying channels of the impacts of the COVID-19 epidemic on distance education patterns. Accordingly, this paper aims to fill this gap and analyzes whether the governmental lockdown measures play any moderating roles in encouraging or discouraging the transforming of the education mode towards distance learning during the COVID-19 pandemic. In addition, existing studies only had a limited perspective on the subject of distance education [8], whereas multiple applications were employed in distance education and their comparisons are less concerned, such as comparing the different COVID-19-induced effects between the traditional and digital appliances in distance learning since school closures. 

In summary, this article attempts to achieve the following research subjects: first, this paper analyzes the influence of the severity of COVID-19 on distance education by applying different applications, including the traditional and digital appliances. Second, it further investigates the impacts of governmental lockdown measures in the linkage between the severity of COVID-19 and the applications that are used in distance learning. Most importantly, based on the empirical results, policy designs are tailored, particularly for developing countries in the process of educational recovery, by transforming towards distance learning patterns in the post-COVID-19 era.

This article mainly has three contributions. First, we further verify the impacts of the severity of the epidemic on distance education by using traditional and digital appliances since school closures. Although there is a general perception that the epidemic has a negative impact on school education, it is empirically unclear whether the COVID-19 epidemic promotes other substitute education patterns, such as distance learning, and in which channels. Second, this paper analyzes the nexus of the mortality rate of COVID-19, governmental blockade policies and distance education by applying different applications. Understanding the associations above is crucial to implement appropriate policies upon educational transformation from the offline learning to distance learning and strengthen resilience to the long-lasting COVID-19 shock. Furthermore, this article combines a number of survey data and investigates 60 developing countries’ governmental and residential responses to COVID-19 to investigate the topics aforementioned.

In particular, this paper employed High Frequency Phone Survey (HFPS) data from a large number of countries with a broader geographical scope, which include a wide variety of social-economic variables during the pandemic. By documenting and analyzing patterns across 60 developing countries across Africa, Asia, South America, North America and Europe, this paper contributes to the growing literature on the heterogeneous impacts of the COVID-19 crisis on distance education in developing countries. The main variables in use contain the county-level percentage of distance education by applying different appliances, such as TVs, radios, and apps, since school closures, the sampled countries’ mortality rate and the infection fatality ratio of the epidemic, and the stringencies of lockdown measures from the authorities. Some other demographic, socio-economic, and geographic factors were also recoded to establish a baseline model. 

In a broad sense, we found strong empirical evidence supporting the positive relationship between the severity of the epidemic and distance learning by applying both traditional and digital appliances. Based on the above analysis, this article calls for affluent policies for the development of distance education in developing countries. In addition, the data in use suggest the gaps/inequality of distance education by applying applications that exist among developing countries. For example, households located in some less developed countries of Asia and Africa, due to a lower level of economic development and larger population, resulted in lower percentages of distance learning since school closures compared with those in the Europe and North America.

The remainder of the paper is structured as follows: Section 2 overviews the literature. Section 3 describes the data in use and the descriptive statistics of the main variables. Then, this section shows the econometric models. The results are reported in Section 4. In Section 5, we summarize the conclusions and provide policy implications.

## 2. Literature Review

The COVID-19 pandemic brought a great shock to each aspect of the society and has been widely discussed in recent studies, such as its adverse effects on economy, health, and employment [17,18,19,20,21]. In particular, worldwide education has also been greatly adversely affected by the global epidemic [15,22,23]. According to the UNESO [24], 1.3 billion students have dropped out from schools since the outbreak of the COVID-19, and more than 150 countries have closed schools in 2020. For instance, Di Pietro et al. [15] emphasized that the stress and anxiety that has arisen from the COVID-19 pandemic have negative impacts on students’ academic performance. Moreover, Onyema et al. [23] noted that this long-lasting pandemic has led to the disruption of offline education and a reduction of education funds and workers, which declines people’s interests in learning. In addition to learning loss, the severe economic impact of the pandemic is expected to increase earlier drop-out, especially among low income households in less-developed countries [25]. 

It is well known that since the outbreak of COVID-19, school closures have been one of the most common measures to protect the health and safety of citizens. Admittedly, this is a risk in facing public health emergencies such as COVID-19 [26]. However, school closures are also the most direct cause of impairing education, particularly during a long-lasting pandemic. First, Jaipuria et al. [27] found that the students’ weekly learning hours during the period of the COVID-19 lockdown were reduced by four to eight hours, indicating the shrinkage of their education hours. Moreover, students’ math and reading skills have declined in various countries due to school closures, suggesting that their learning ability is weakened [6]. School closures also lead to a reduction in various academic activities, such as seminars and conferences [28]. Second, the shutting down of schools has led to more than one fifth of the students not undertaking exercise in the past two weeks [26]. Third, the epidemic further aggravates the inequality of education among different countries. Lorente et al. [29] claimed that developed countries can better deal with the social crisis compared with developing ones, and hence the educational inequality will even become more remarkable in the post-crisis phase. Furthermore, educational inequality is likely to trigger other social inequalities. For example, gender equality is threatened in developing countries because of school closures as more than 10 million girls will be dropped out from school and are at risk of early marriage in the next decade [6]. The above literature suggests that, on the one hand, countries tend to close schools to prevent the spread of COVID-19; on the other hand, this measure sacrifices children’s education. In summary, a growing body of literature has confirmed the negative effects of the pandemic on formal education due to school closures, but fewer studies have explored its effects on distance learning, which is a substitute of formal education. 

Distance education is a potential system to provide extra educational chances when the formal one has to be stopped. The conception of distance education originally took place via the postal service in the USA in the 18th century [30], and involved interactive telecommunications systems to connect learners, resources, and instructors [31]. Garrison [32] indicated that the development of distance education had three generations of technology, namely correspondence, telecommunications, and computer. In the modern society, online education is becoming a prevalent way to ensure the smooth process of teaching without physically getting together [33]. Several advantages of distance learning by applying applications have been well explored in the existing literature. Ebuete et al. [34] proved that online education, a typical type of distance education, enables teachers and students to realize effective learning through using digital applications [35] Additionally, distance education allows for continual teaching and learning without interruptions [36,37]. Therefore, distance education is without the limitation of geographic restrictions and even extends to other countries around the world, and hence it is more likely to optimize resources [38,39]. Emergency remote education (ERE) is another branch of distance education which plays an important role in crises [13]. ERE is defined as the temporary transition of instructional delivery to an online delivery model due to a sudden crisis [40]. It differs from distance education in that ERE provides interim teaching support in a rapidly established way, and it is only reliably delivered during emergencies or crises [41]. 

Some studies have begun to recognize people’s participating in distance learning during the COVID-19 pandemic, and 68% of the 127 countries are employing both digital and non-digital applications in order to access distance education. Those applications that were in use during the pandemic contain televisions, radios, and take-home packages [42]. Meanwhile, a survey highlighted that most college students were satisfied with distance education [43,44]. However, developing distance learning worldwide poses challenges to some regions as well. Existing evidence shows that at least a third of the world’s school children do not have access to distance learning after school closures as the local areas or families lack relative policies or equipment to support their learning at home [15,45]. In fact, the technology gap between developed and developing countries exacerbates the inequality in distance education opportunities [29]. Yet many of the children—particularly those in poorer households—do not have internet access, personal computers, TVs, or even a radio at home, amplifying existing education inequalities between richer and poorer countries and between better-off and worse-off households within those countries. To address these issues, Ferri et al. [46] proposed that governments should use diverse modalities to provide accessible learning experiences for students in remote areas. For instance, governments could reduce internet costs for poorer households and even guarantee a free provision of computers and laptops for students in need [15]. Overall, the role of distance education during the COVID-19 pandemic is crucial, and its potential role could be larger if the society makes more efforts in this pattern of education in the near future.

Besides the requirements of school closures, governments and authorities have implemented a wide variety of interventions to contain the spread of the pandemic, such as imposing stay-at-home orders, forbidding travelling, and taking personal hygiene measures such as wearing masks and washing hands [21]. On the one hand, those restriction policies are effective in alleviating the severity of the pandemic. Okuonghae and Omame [47] demonstrated that if at least 55% of the population keeps a social distance and uses masks effectively in public, this could lead to a significant reduction in the number of the confirmed COVID-19 cases; stricter restrictions on people in high-risk regions are more helpful in slowing down the spread of COVID-19 [48,49]. Similarly, Piovani et al. [50] showed that the decisions of school closures during COVID-19 outbreaks are associated with significant reductions in cumulative COVID-19 deaths. On the other hand, however, strict lockdown measures do not play significant roles in some specific situations. Haider et al. [51] noted that in Africa’s vast and dense settlements, lockdown measures may even increase the spread of COVID-19. Melnick et al. [52] argued that lockdown that was imposed during highly contagious activities could force infected people to spend more time in confined spaces with relatives, which would lead to an increase in infection rates. However, the impacts of pandemic lockdown measures on distance learning are without empirical results as yet.

In light of the above-mentioned literature, it is apparent that the COVID-19 pandemic has a negative impact on formal education due to school closures. Distance education by applying applications is becoming an alternative pattern to ensure the process of education worldwide. However, few studies have explored the effects of COVID-19 on distance education. Moreover, the roles of lockdown measures are largely ignored in determining distance learning as well. If, however, the lockdown requirements’ moderating the effects in the association between the severity of the COVID-19 pandemic and distance learning are further examined, timely policies can be given to guide the transformation of education patterns during the global epidemic, which is crucial to continue the basic education of millions of children. Thus, our work aims to fill the above gaps.

## 3. Data and Methodology

This section first introduces how we combined different data sources in terms of establishing a rich dataset to investigate the impacts of the COVID-19 epidemic on education by employing various applications. Then, this section describes the main variables that were used in the empirical models and shows the patterns of the main variables and their potential linkages. Ultimately, the methodology subsection establishes the baseline empirical models to present the associations above. Furthermore, the underlying moderating effects of the lockdown measures that are required by the authorities are explored in the linkage between the mortality rate of COVID-19 and distance learning by applying different applications.

### 3.1. Data

**Figure 1:**
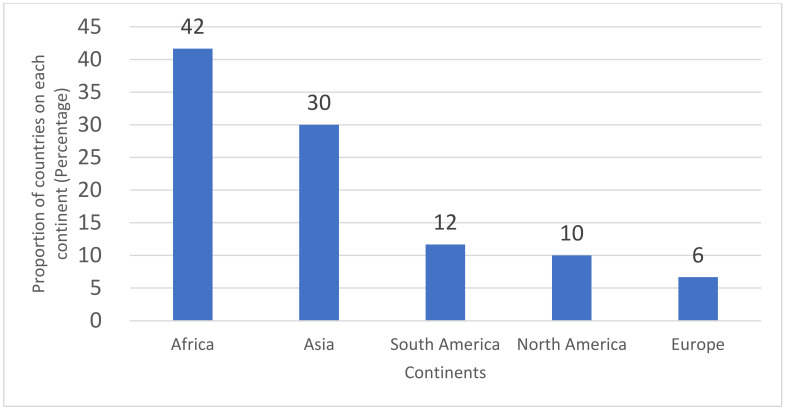
Continental distribution of the 60 sampled countries.

Broadly speaking, there are four data sources that were employed in this study. The first data in use, named HFPS (data accessed from: https://www.worldbank.org/en/data/interactive/2020/11/11/covid-19-high-frequency-monitoring-dashboard, accessed on 29 October 2021), is about various socio-economic variables in response to the COVID-19 pandemic on households and/or individuals across a wide variety of developing countries. The data version of 29 October 2021 is the latest available version that was applied for this research, and it covers the recodes of 83 developing countries in total. This study focused on the adoption of applications in distance learning since the shutting down of schools due to the COVID-19 epidemic. The variables of interest are a series of children’s education models through various applications since school closures. To be specific, those variables contain the country-level rate of children’s learning through traditional appliances, including the TVs and radios, and through the digital ones, i.e., the apps of the sampled households. By combining the above two categories of applications for distance learning, a proportion of using comprehensive appliances for children can be obtained for each of the sampled countries. There were 60 sampled countries that were distributed in six continents that included the information of the applications that were employed in distance learning since school closures. The distribution of those countries is as follows: there were 25 of the total samples that were located in Africa, and 18 came from Asia. The remaining 17 countries were from South America, North America, or Europe. Those that were from Europe were the least with only 7% in total. It is clear that the sampled countries were mainly from the least developed regions of the world (see Figure 1).

**Figure 2:**
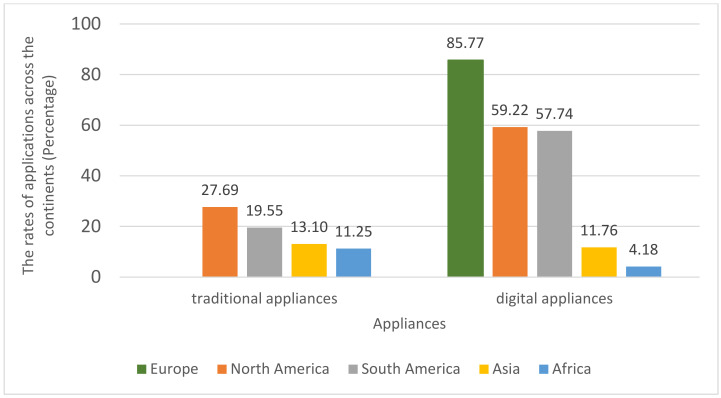
The rates of traditional applications and modern applications across the continents.

Figure 2 compares the uses of traditional and digital applications by continents. Due to the different levels of economic development, the use of appliances for distance education also varies across countries and continents. It is clear that the European sampled households on average have the highest percentage in employing digital applications with a percentage of 85.77%. This was followed by the samples in North America with 59.22%, South America (57.74%), and Asia (11.76%). Africa had only 4.18% of the sampled households apply digital applications in distance learning. It can be seen that the rate of digital appliances in European and American households are much higher than those in Asia and Africa. In terms of the rate of traditional appliances usage, the absolute percentages are comparatively smaller than the modern ones except for African countries with a proportion of 11.25%. Similarly, the proportions of traditional applications in distance learning in North and South America still surpass those in Asia and Africa (European countries do not have available records for traditional applications, and therefore the corresponding percentage does not appear in the figure). In the HFPS data, the school attendance rates in European and American countries are much higher compared with Asia and Africa. Hence, Figure 2 illustrates that distance education by applying applications since the shutting down of schools is more widely implemented in regions with higher levels of economic development and educational quality. It is worth noting that this figure only presents the records of the sampled countries, and did not have continental representativeness. Nevertheless, it is evidence of the applications usage of the households that are located in different continents and is in line with the economic and educational levels.

Some other demographic variables that were included in the HFPS were also used as controls in the empirical models. Those variables are the school attendance rate before school closures and some family characteristics including the average family size and the percentage of male respondents. Furthermore, the interviewing time was also included in order to consider the time effect.

The HFPS data were designed to be nationally representative. Although specific procedures differ by country, all datasets have been reweighted to adjust for differential response rates among the subgroups of populations, with the objective of obtaining estimates as close to nationally representative as possible. Notably, as households without phones are not achievable, their educational modes by applying various applications could be even less. Still, the effects of COVID-19 on distance learning by applying applications can be examined based on random selections from households owning phones at home, although the impacts could be overestimated particularly in the least developed regions.

**Figure 3:**
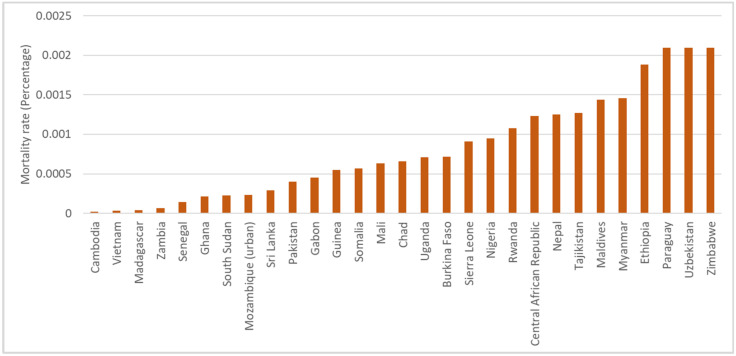
A total of 29 countries’ mortality rates of the COVID-19 epidemic that were lower than the median of the whole sample.

**Figure 4:**
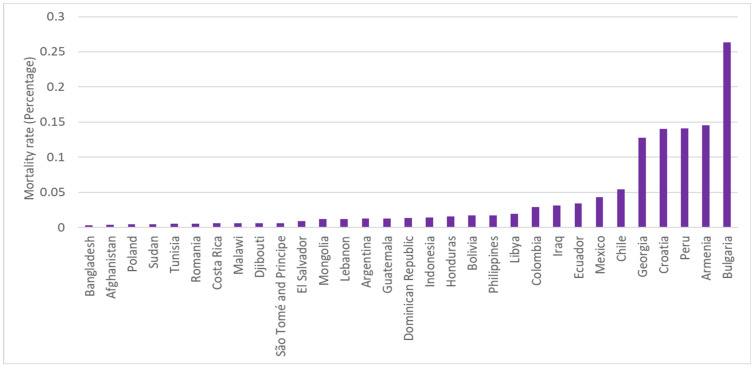
A total of 31 countries’ mortality rates of the COVID-19 epidemic that were higher than the median of the whole sample.

To quantify the severity of COVID-19 for the above-mentioned 60 countries, we follow Le et al. [53] to choose the mortality rate of the corresponding country at the interviewing month. In addition, the infection fatality ratio (IFR) is another indicator of the severity of the COVID-19 epidemic. Those rates are downloaded from the COVID-19 Data Repository by the Center for Systems Science and Engineering (CSSE) at Johns Hopkins University (JHU CSSE COVID-19 Data (data accessed from: https://www.worldbank.org/en/data/interactive/2020/11/11/covid-19-high-frequency-monitoring-dashboard, accessed on 29 February 2022). The data covered the records of 216 countries in total. In practice, we matched the rates of the sampled countries to the interviewing time of the HFPS and obtained the corresponding month rates for each sample. A higher percentage of the rate represents that the epidemic is more severe within the country during the interviewing period. Figure 3 and Figure 4 show the ordered mortality rates across the countries by separating the samples into the lower and higher mortality rate groups.

The countries in Figure 3 are those with a mortality rate that was lower than the median value of the whole sample. To be specific, the mortality rate of Cambodia, Vietnam, Madagascar, and Zambia were lower than the others, with the lowest mortality rates of 0.00002% in Cambodia.

In Figure 4, the mortality rate varied widely across the 31 countries. In particular, the mortality rate of Bulgaria, Armenia, Peru, Croatia, and Georgia were much higher than the others, with mortality rates of 0.26%, 0.15%, 0.14%, 0.14%, and 0.13%, respectively. The number of deaths per million in the top five sampled countries varied from 1000 to 3000, which is much higher than the mortality rates among other countries. For example, the mortality rate per million in China and India was only 3 and 41.5, respectively. Even in Italy, the deaths per million was 586, which is lower than the five countries that are mentioned above [54].

The third database in use contained the stringency of lockdown measures. Accordingly, the Oxford COVID-19 Government Response Tracker (OxCGRT (data accessed from: https://www.bsg.ox.ac.uk/research/research-projects/covid-19-government-response-tracker, accessed on 11 March 2022) systematically collects publicly available information on 21 indicators of several COVID-19 containment measures, such as the lockdown measures and the stay-at-home orders, that governments take in response to the pandemic. The three variables about the lockdowns were restricted to at home, at the workplace, and in traffic. The scores of blocking at home and restrictions on the workplace ranged from 0 to 3, while the traffic restriction term ranged from 0 to 2. Higher scores represent stricter policies on the containment.

Eventually, some other socio-economic variables were taken from the World Bank (Data accessed from: https://data.worldbank.org/, accessed on 11 March 2022) to match with the HFPS, including the county-level old age ratio, foreign direct investment (FDI), and GDP. The definitions of all the main variables are defined in Table 1, while Table 2 offers some descriptive statistics of these variables.

**Figure 5:**
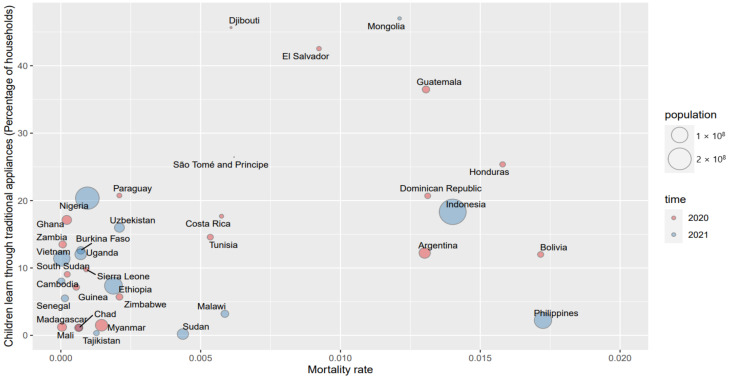
The pattern of the mortality rate and traditional appliances in children’s education since school closure.

**Figure 6:**
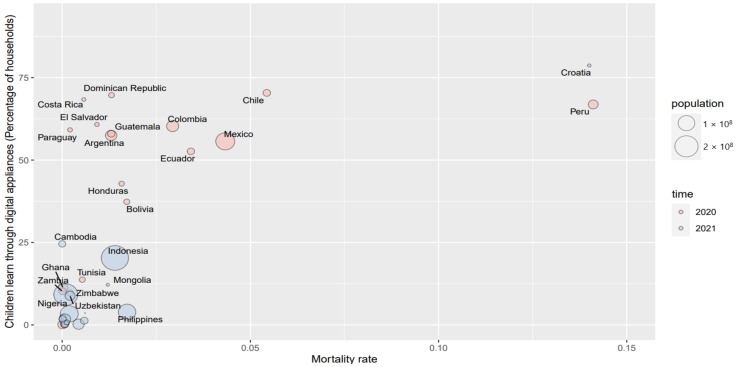
The pattern of the mortality rate and digital appliances in children’s education since school closure.

**Figure 7:**
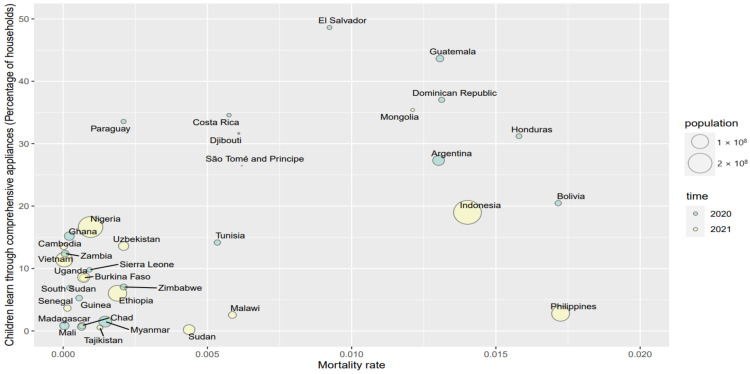
The pattern of the mortality rate and comprehensive appliances in children’s education since school closures.

In order to further understand the patterns between the COVID-19 mortality rate and the children’s alternative education by applying various applications since school closures, Figure 5, Figure 6 and Figure 7 briefly illustrate those associations.

In Figure 5, Figure 6 and Figure 7, the X axis is the mortality rate and the Y axis represents the different appliances that were employed in distance education since school closures, including traditional appliances, digital appliances, and comprehensive appliances, respectively. The sizes of the bubbles are the countries’ population.

According to Figure 5, the mortality rate is generally positively correlated with the traditional appliances when the mortality rate is lower than 0.015%, which indicates that the severity of epidemic contributes to distance learning by applying traditional medias. An increase in the mortality led to the closure of more schools and thereby distance education emerged. TVs and radios were the most common applications, therefore the pattern of the mortality rate and traditional appliances in distance learning has a positive trend for countries with lower mortality rates. However, there are also countries with higher rates of mortality (such as those with a mortality rate that is higher than 0.015%) but an even lower usage of traditional media. This phenomenon suggests that the pattern between the two issues is more complex and should be further explored by considering many other socio-economic and demographic variables.

Overall, the mortality rate has a clear positive correlation with distance education by applying digital appliances in Figure 6. Similar to Figure 5, some exceptions exist in the graph: some countries with low mortality also adopt the digital applications in distance learning, such as Costa Rica, with almost 74% in digital appliances, but it does not experience a significantly higher mortality rate.

By combining traditional and digital applications, distance education by employing comprehensive appliances is more prevalent in higher mortality rate countries, although there is variation (see Figure 7). For example, the Philippines has a high mortality rate, but the comprehensive appliances rate is only 2.80%.

In sum, according to the harmonized dataset and the plotted figures, the COVID-19 mortality rate varies across countries and it has a potential positive correlation with distance learning by applying different sorts of appliances. Meanwhile, there are some fluctuations which suggest that the linkages between the two issues should be further placed in the empirical models.

### 3.2. Methodology

This subsection mainly presents the empirical models in order to examine the impacts of the severity of COVID-19 on distance learning by applying various applications in this empirical research.

First, we employed four data surveys that were aforementioned and obtained a harmonized dataset through the method of data matching. In the empirical investigation process, we used the robust ordinary least squares (OLS) as the baseline regression models to empirically examine the aforementioned associations. The robust OLS model is capable of reflecting the causal relationship between the key variables.

(1) $$TA_{i}=\;+\;\alpha_{0}\;+\;\alpha_{1}{Mortality}_{i}\;+\;\alpha_{2}X_{i}\;+\;\delta_{i}\;+\;\varepsilon_{i}\;$$

(2) $$DA_{i}\;=\;\beta_{0}\;+\;\beta_{1}{Mortality}_{i}\;+\;\beta_{2}X_{i}\;+\;\eta_{i}\;+\;\mu_{i}\;$$

(3) $${CA}_{i}\;=\;\gamma_{0}\;+\;\gamma_{1}{Mortality}_{i}\;+\;\gamma_{2}X_{i}\;+\;\omega_{i}\;+\;\theta_{i}\;$$

In Equation (1), where *TA_i_* represents the rate of distance learning through TVs and radios for country *i*. *Mortality* denotes the country-level mortality rate for country i in the interviewing month. $\alpha_{0}$ is the constant. *X* contains various control variables and continental dummies that were mentioned in the data subsection. $\delta_{i}$ is the fixed effect term of the interviewing time. $\varepsilon_{i}$ is the error term. Similarly, *DA* in Equation (2) represents the percentage of distance learning through apps. *CA* in Equation (3) is the rate of learning through the combining of traditional and digital appliances.

Then, we examined the moderating effects by analyzing whether the linkage between the mortality rate and distance learning by applying different applications has a channel through the stringency of governmental lockdown measures.

The reason for applying the moderating effects in this article is that they are applicable in testing the underlying mechanisms of the linkage between the mortality rate and distance education by applying different applications. In particular, this article explores the channel of governmental lockdown measures to establish the nexus. Accordingly, we estimate Equations (4) and (5) for traditional and digital applications that are used in distance learning, respectively:

(4) $${TA}_{i}\;=\;\alpha_{0}\;+\;\alpha_{1}{Mortality}_{i}\;+\;{Mortality}_{i}{\times Lock}_{i}\;+\;Lock_{i}+\alpha_{2}X_{i}+\delta_{i}\;+\;\varepsilon_{i}\;$$

(5) $${\;DA}_{i}\;=\;\beta_{0}\;+\;\beta_{1}{Mortality}_{i}\;+\;{Mortality}_{i}{\times Lock}_{i}\;+\;Lock_{i}+\beta_{2}X_{i}\;+\;\eta_{i}\;+\;\mu_{i}$$

${Lock}_{i}$ includes the measures taken to contain at home (Home), blockade of the workplace (Work), restrictions on traffic (Transport) and a comprehensive lockdown index by combining the three lockdown measures above. Where ${Mortality}_{i}{\times Lock}_{i}$ represents the interaction term of the mortality rate and the different aspects of lockdowns. These lockdown policies could be helpful in controlling the spread of COVID-19, but they could also bring the extra cost of the holding of offline education. Hence, it is necessary to examine the nexus of the severity of COVID-19, the containment policies, and distance education by employing traditional and digital applications. The results are important in guiding policy implications with regard to promote distance education by using applications in the post-crisis phase [55].

## 4. Results

This section presents and discusses the main regression results based on the methodology part. First, the baseline regression results are reported to show how the severity of the COVID-19 epidemic affects the use of appliances in distance education since school closures. Then, the regulations upon lockdown are added in the model to explore the underlying mechanisms of the associations above.

### 4.1. The Baseline Regression Results

Table 3 reports the regression results for Equations (1)–(3) where the dependent variables are children’s education by employing traditional appliances, digital appliances, and comprehensive appliances, respectively. The core results of the mortality rate on application usage are significantly positive across the models. Columns (1), (3), and (5) of Table 3 reports the empirical results without many controls, while the left three columns include extra control variables such as the interviewing time and continental dummies to examine the robustness of the linkage. Broadly speaking, some other terms are as expected, i.e., GDP and education rate before school closures are positively significant with distance education by applying applications. The signs are consistent with the World Bank, UNESCO, and UNICEF (2021) that high income countries are better equipped with resources to encourage distance learning with wider appliances. Conversely, a larger population of a country and larger family size leads to lower rate of distance learning.

Specifically, the coefficients before the mortality rate differ between the columns of traditional and digital appliances (ranging from 66.611 for digital applications to 215.759 for traditional applications). Apparently, the coefficients before the variable of mortality rate are smaller for distance learning by applying digital appliances. This suggests that the mortality rate of COVID-19 promotes distance learning by using traditional applications which is more common in developing countries, and the results are coincided with common sense, as traditional applications are more available in countries with a lower level of economic and social development.

Under a similar setup, Table 4 reports the empirical results using the infection fatality ratio (IFR) as another proxy for the severity of COVID-19. The results are generally consistent with Table 3 which proves the reliability of the baseline results. There is one exception that the IFR is without any statistically significance before the term of the digital appliances when all the variables are controlled. Similarly, the GDP, population, and household size are significant to distance education by employing appliances.

It is worth noting that compared with the severity of COVID-19, the mortality rate has stronger effects both in terms of the level of significance and magnitude. These differences suggest that the mortality rate of COVID-19 has stronger and broader impacts on the educational mode by employing appliances.

### 4.2. The Regression Results of Moderating Effects

Governmental lockdown measures have been rarely included as an underlying channel in understanding the shock of COVID-19 on distance education. We expect that stricter lockdown measures tend to hinder the promotion of educational mode by employing various appliances in the COVID-19 crisis. Accordingly, Table 5 reports a series of regression results with the moderating effects of lockdowns by adding interaction terms of the severity of COVID-19 and the lockdown measures.

For the terms of using traditional appliances, we found significant roles stemming from restricted at home, restricted on the workplace, and traffic. A stricter lockdown policy was associated with a lower rate of applying traditional appliances, whereas it had no significant impact on the employment of digital appliances. The interaction terms of the mortality rate and lockdown policies were also negative for using the traditional appliances. The reason behind this could be that a stricter lockdown measure tends to interrupt the work progress of TV and radio programs. Consequently, children do not have many choices of educational TV and radio programs. Conversely, the pandemic containment measures do not have any significant effects on the application of digital appliances as apps and internet are comparatively less affected by the lockdown measures upon workers and traffic. Fuller et al. [52] also proved that the government’s lockdown measures would lead to the interruption of education. Bundervoet et al. [8] also suggested that the lockdown measures from the authorities could have a negative impact on distance education, but this literature did not specify distance learning by using representative and various appliances for analysis.

## 5. Conclusions and Policy Implications

Distance learning by employing various appliances is crucial for global development in a wave crisis such as the long-lasting COVID-19 pandemic. The shutting down of schools has widened learning inequalities and has harmed the education chances of children around the world, especially in low-income developing countries, where the resources of education are quite limited and education inequality are more common. Following a large body of literature that examined the effects of the pandemic on education, our work mainly sheds light on a substitutable education pattern, the distance learning with different applications, and takes a comprehensive perspective to investigate how the severity of the COVID-19 pandemic in developing countries affects distance education by employing various appliances. By merging a couple of data resources, we obtained data from 60 developing countries across five continents and recorded their household-based distance education by employing various applications after the shutting down of schools due to COVID-19. Continental heterogeneities are well observed: European and American households had a much larger percentage of applying digital applications in distance learning comparing with their African counterparts. Households that were located in African countries tended to employ traditional applications instead of digital ones. Many other social, political, and economic variables were included to establish the associations between the severity of COVID-19 and distance learning. Our main findings showed that the COVID-19 pandemic has positive impacts on distance learning by applying different applications, such as TVs, radios, and apps.

Although a number of previous studies have recognized that many countries began to adopt distance learning in response to school closures since the COVID-19 pandemic, our research has expanded this direction of research by further exploring the moderating role of different lockdown regulations on the association between the mortality rate of COVID-19 and distance learning by applications. The empirical results suggest that those lockdowns negatively affected distance learning by using traditional medias, including TVs and radios, in developing countries. However, this effect was not significant for the applications of using apps. Thus, extensions from the negative effects of the pandemic on education to the modes of how technological, economic, political, and many other aspects could prevent the disruption of children’s alternative education are crucial in the post-crisis phase. In this article, we discovered that policies need to focus on fostering an inclusive educational recovery and governments should take actions to popularize distance learning by applying multi-appliances to strengthen resilience for future shocks. The above-mentioned suggestions are of great significance to resolve the potential educational crisis and obtain a long-term social sustainability.

Based on the empirical results, a number of specific policy implications can be summarized particularly aiming at the post-crisis phase: first, it is important for policy-makers to further promote distance learning by applying various applications in response to the COVID-19 pandemic. Nevertheless, schools have to be closed for a particular period of time during the COVID-19 crisis. The policy implications of this article coincide with Ferri et al. [43], which noted the complemented role of the online lessons when the face–face lessons are not available. Second, policy designs should encourage diversity of distance learning. For instance, the modes of distance learning can extend to employ various resources and applications. Both digital technologies and traditional medias can be implemented to enhance the possibilities of carrying out distance learning. Teachers, educators, and trainers should be frequently encouraged by governments to contribute to transform from offline education to distance learning during the COVID-19 pandemic. In particular, some developing countries in Asia and Africa should promptly transform their education models to reduce their educational inequality gap with high-income countries. Third, local governments should aid poorer households to access useful educational appliances. Countries with large populations need to take timely actions to reduce the cost of applying appliances. For example, governments can enrich fundamental structures to reduce internet costs, which further popularize distance learning through fast speed of WiFi or stable internet. Fourth, in addition to caring for health issues, policies should focus on broader aspects and prepare for other types of disasters and future crises, particularly among the vulnerable and the poor, which are capable of enhancing the well-being of citizens [56]. In response to school closures, other basic services should keep up with the education policies, such as providing informal childcare and supports [57].

From the perspective of schools and other institutions, schools should provide systematic training initiatives to improve teachers’ technology skills, and hence, they can timely respond to such shutting down emergencies. In addition, international organizations should provide education assistance to the low-income developing countries. For example, UNESCO and France appealed a global initiative to increase investment in education in the aftermath of the COVID-19 crisis [58].

Our research is based on a harmonized dataset with a broader geographic scope of 60 developing countries. To some extent, the results are capable of shedding light on some implications for other emerging economies as well. The dilemma of distance learning by applying multi-appliances, particularly digital ones, is not unique to these least developed countries from the broader Africa and Asia, and they are also relevant to most emerging economies and even to some developed countries. Thus, it is necessary to explore the relative research topic in a broader sense. In addition, based on the systematic literature review, we recognize that different categories of distance education, such as ERE and online distance learning, is particularly important for different stages of a crisis. In this sense, distance education can be further identified and explored based on local specifics.

The limitations of this article are mainly in the following three aspects: first, this article only focused on the traditional and digital applications in distance learning. Apparently, the understanding of implementing distance learning modes is far from decent. Many other issues upon alternative education patterns should be taken into account, such as educational resources, measures, organizers, and regulations for guaranteeing a wide variety of informal learning to deal with a sudden and even a persistent shock. Second, this paper adopts cross-sectional data which cannot measure the long term impacts of COVID-19 on alternative education. Moreover, due to the limitation of data sources, this article descriptively analyzes distance education at the macrolevel, which is insufficient to dig out detailed information of the behaviors/decisions of governments and households in facing the pandemic. Thus, more studies are needed to focus on the panel and/or microlevel data, and this might reveal many other interesting findings based on the current research direction. Third, due to different economic and demographic factors, the results of this article might be differently presented in other developing economies where, for instance, the COVID-19 pandemic is well controlled and educational resource is affluent, i.e., in China, and among well-developed countries.

## Figures and Tables

**Table 1:** Descriptions of the main variables.

Variable	Definition	Data Source
Mortality	The country-level mortality rate in the interviewing month	COVID-19 Data Repository by the Center for Systems Science and Engineering (CSSE) at Johns Hopkins University (JHU CSSE COVID-19 Data)
IFR	Infection fatality ratio	
Population	The natural logarithm term of population	
Africa	Households located in Africa (“1” true, “0” false)	
Asia	Households located in Asia (“1” true, “0” false)	
Europe	Households located in Europe (“1” true, “0” false)	
North America	Households located in North America (“1” true, “0” false)	
South America	Households located in South America (“1” true, “0” false)	
TA	The rate of children’s distance learning by applying TVs and radios since school closures	High Frequency Phone Survey (HFPS)
DA	The rate of children’s distance learning by applying apps since the school closures	
CA	The rate of children’s distance learning by applying TVs, radios, and apps since school closures	
Edu	The school attendance rate of children before school closures	
Male	The natural logarithm term of the rate of the male respondents	
Size	The number of household members	
Time	The interviewing time (ranged from May 2020 to August 2021. In empirical analysis, numbers such as 202,005 are used to represent the interviewing time of May 2020)	
GDP	The natural logarithm term of GDP (US dollars) in the year 2019	World Bank
FDI	The natural logarithm term of the net inflow of foreign direct investment (US dollars)	
Aging	The natural logarithm term of the percentage of the population with the age older than 64 to the population within the age ranging from 15 to 64.	
Home	The stringency of stay-at-home orders (varies from “0” very free to “3” very strict, integers only)	Oxford COVID-19 Government Response Tracker (OxCGRT)
Work	The stringency of blockade to the workplace (varies from “0” very free to “3” very strict, integers only)	
Transport	The stringency of lockdown of the traffic (varies from “0” very free to “2” very strict, integers only)	
Lockdown	The comprehensive lockdown index which includes blockade at home, to the workplace, and in traffic. (it is a normalized value ranging from 0 to 1; varies from “0” very free to “1” very strict)	

**Table 2:** Descriptive statistics of the main variables.

Variable	Obs	Mean	Std. Dev.	Min	Max
Mortality	60	0.0002	0.0005	1.77 × 10^−7^	0.0026
IFR	60	0.024	0.024	0.001	0.137
TA	40	0.155	0.128	0.002	0.47
DA	38	0.282	0.294	0.001	0.929
CA	42	0.215	0.206	0.002	0.929
GDP	59	24.492	1.590	19.873	27.870
Population	60	16.660	1.389	12.317	19.437
Edu	47	0.803	0.186	0.228	0.999
Male	60	−0.630	0.219	−1.077	−0.095
Size	53	4.984	1.638	2.311	10.379
FDI	54	20.536	1.762	16.378	24.159
Aging	60	−2.469	0.558	−3.265	−1.090
Home	56	1.388	0.958	0	3
Work	56	1.595	0.805	0	3
Transport	56	0.711	0.691	0	2
Lockdown	56	0.450	0.260	0	1
Time	60	202,041	46.047	202,005	202,108
Africa	60	0.417	0.497	0	1
Asia	60	0.3	0.462	0	1
Europe	60	0.067	0.252	0	1
North America	60	0.1	0.303	0	1
South America	60	0.117	0.324	0	1

**Table 3:** The associations of the mortality rate and different sorts of appliances that were applied in distance education by using the robust OLS regression.

	TA	TA	DA	DA	CA	CA
Mortality	141.296 **	215.759 ***	115.755 ***	66.611 **	205.020 ***	151.828 ***
	(61.420)	(52.327)	(40.656)	(25.104)	(17.488)	(32.006)
GDP	0.055 **	0.101 **	0.182 ***	0.024	0.090 ***	0.075 **
	(0.027)	(0.042)	(0.027)	(0.037)	(0.018)	(0.031)
Population	−0.093 ***	−0.164 ***	−0.195 ***	−0.054	−0.126 ***	−0.128 ***
	(0.031)	(0.048)	(0.044)	(0.032)	(0.021)	(0.030)
Edu	0.012	−0.078	0.488 ***	0.094	0.143 **	−0.022
	(0.075)	(0.093)	(0.118)	(0.112)	(0.053)	(0.061)
Male	0.029	0.113	0.050	0.142*	0.053	0.111
	(0.096)	(0.090)	(0.131)	(0.073)	(0.064)	(0.067)
Size	−0.002	−0.019 *	−0.024 **	−0.018 **	−0.012 *	−0.018 **
	(0.011)	(0.010)	(0.011)	(0.007)	(0.006)	(0.007)
FDI		0.011		0.024		0.016
		(0.024)		(0.018)		(0.016)
Aging		−0.155 *		0.056		−0.094
		(0.090)		(0.050)		(0.063)
Time		0.001		0.000		0.001
		(0.001)		(0.000)		(0.000)
Constant	0.384	−155.267	−1.203 **	−52.197	0.047	−111.716
	(0.284)	(101.870)	(0.532)	(81.790)	(0.200)	(70.648)
Continent dummies	NO	YES	NO	YES	NO	YES
N	38	35	36	34	40	37
Adj.$R^{2}$	0.382	0.458	0.824	0.968	0.874	0.909

Note: (1) Standard errors in parentheses; * *p* < 0.1, ** *p* < 0.05, *** *p* < 0.01; (2) “YES” means controlling continental geographical dummies, while “NO” means the geographical dummies are not included in.

**Table 4:** The associations of the infection fatality ratio and different sorts of appliances that were applied in distance education by using the robust OLS regression.

	TA	TA	DA	DA	CA	CA
IFR	0.959	1.780 **	1.971 ***	0.102	1.400 **	1.311 *
	(0.705)	(0.762)	(0.565)	(0.563)	(0.547)	(0.635)
GDP	0.064 **	0.119 **	0.187 ***	0.020	0.130 ***	0.077 **
	(0.026)	(0.043)	(0.026)	(0.036)	(0.028)	(0.033)
Population	−0.103 ***	−0.197 ***	−0.220 ***	−0.052	−0.177 ***	−0.145 ***
	(0.031)	(0.047)	(0.040)	(0.031)	(0.032)	(0.030)
Edu	−0.016	−0.147	0.478 ***	0.058	0.163 *	−0.070
	(0.093)	(0.095)	(0.133)	(0.120)	(0.088)	(0.068)
Male	0.020	0.134	0.119	0.151 *	0.177	0.140 **
	(0.098)	(0.083)	(0.115)	(0.075)	(0.108)	(0.062)
Size	−0.002	−0.026 **	−0.038 **	−0.019 **	−0.028 *	−0.026 ***
	(0.011)	(0.010)	(0.016)	(0.007)	(0.016)	(0.007)
FDI		0.015		0.028		0.024
		(0.023)		(0.018)		(0.016)
Aging		−0.179 *		0.057		−0.108
		(0.095)		(0.048)		(0.069)
Time		0.001 **		0.000		0.001 **
		(0.001)		(0.000)		(0.000)
Constant	0.334	−223.141 **	−0.821	−59.197	0.090	−168.522 **
	(0.291)	(101.596)	(0.588)	(88.198)	(0.362)	(71.258)
Continent dummies	NO	YES	NO	YES	NO	YES
N	38	35	36	34	40	37
Adj. R^2^	0.361	0.449	0.826	0.963	0.715	0.894

Note: (1) Standard errors in parentheses; * *p* < 0.1, ** *p* < 0.05, *** *p* < 0.01; (2) “YES” means controlling continental geographical dummies, while “NO” means the geographical dummies are not included in.

**Table 5:** The robust OLS regression results by adding the lockdown measures.

	TA	DA	TA	DA	TA	DA	TA	DA
Mortality	448.371 ***	64.103 **	331.908 ***	33.241	531.454 ***	66.413 **	462.491 ***	66.944 **
	(69.135)	(29.475)	(65.209)	(56.086)	(106.629)	(26.772)	(78.138)	(25.456)
Home	−0.053 **	0.010						
	(0.022)	(0.013)						
Mortality × Home	−359.591 ***	−13.986						
	(100.547)	(20.620)						
Work			−0.054	−0.018				
			(0.032)	(0.030)				
Mortality × Work			−311.833 **	51.905				
			(136.188)	(43.526)				
Transport					−0.011	0.001		
					(0.042)	(0.017)		
*Mortality* × Transport					−611.076 ***	−5.227		
					(193.534)	(39.294)		
Lock average							−0.084	0.027
							(0.093)	(0.047)
*Mortality* × *Lock* average							−1195.194 ***	−30.648
							(349.896)	(124.807)
GDP	0.096 **	0.025	0.097 **	0.021	0.125 **	0.025	0.102 **	0.026
	(0.043)	(0.038)	(0.044)	(0.043)	(0.045)	(0.044)	(0.045)	(0.041)
Population	−0.169 ***	−0.058 *	−0.162 ***	−0.047	−0.223 ***	−0.055	−0.185 ***	−0.060
	(0.046)	(0.032)	(0.053)	(0.039)	(0.046)	(0.041)	(0.048)	(0.037)
FDI	0.018	0.024	0.021	0.024	0.024	0.024	0.021	0.024
	(0.023)	(0.019)	(0.024)	(0.020)	(0.022)	(0.019)	(0.022)	(0.019)
Male	0.166	0.168 **	0.102	0.116	0.305 ***	0.145	0.203 *	0.161 *
	(0.102)	(0.078)	(0.095)	(0.083)	(0.102)	(0.085)	(0.110)	(0.087)
Size	−0.022 *	−0.019 ***	−0.017	−0.023 **	−0.009	−0.018 *	−0.015	−0.017 **
	(0.011)	(0.006)	(0.012)	(0.010)	(0.012)	(0.009)	(0.012)	(0.007)
Edu	−0.071	0.080	−0.074	0.130	−0.150	0.090	−0.084	0.077
	(0.092)	(0.120)	(0.088)	(0.142)	(0.091)	(0.131)	(0.090)	(0.133)
Aging	−0.154 *	0.058	−0.156	0.046	−0.139	0.056	−0.145	0.058
	(0.088)	(0.047)	(0.093)	(0.052)	(0.084)	(0.052)	(0.086)	(0.050)
Time	0.001	0.000	0.001	0.000	0.001 *	0.000	0.001 *	0.000
	(0.000)	(0.000)	(0.001)	(0.001)	(0.001)	(0.000)	(0.001)	(0.000)
Constant	−167.095	−68.168	−143.344	−12.699	−238.272 *	−53.439	−208.257 *	−65.600
	(99.254)	(81.882)	(105.630)	(110.395)	(121.055)	(88.767)	(113.815)	(92.263)
Continent dummies	YES	YES	YES	YES	YES	YES	YES	YES
N	35	34	35	34	35	34	35	34
$\mathrm{Adj}.\;R^{2}$	0.489	0.966	0.462	0.965	0.568	0.964	0.504	0.964

Note: (1) Standard errors in parentheses; * *p* < 0.1, ** *p* < 0.05, *** *p* < 0.01; (2) “YES” means controlling continental geographical dummies, while “NO” means the geographical dummies are not included in.

## Data Availability

The dataset generated and/or analyzed during the present study is available from the corresponding author.
